# *Phomopsis longanae* Chi-Induced Changes in Activities of Cell Wall-Degrading Enzymes and Contents of Cell Wall Components in Pericarp of Harvested Longan Fruit and Its Relation to Disease Development

**DOI:** 10.3389/fmicb.2018.01051

**Published:** 2018-05-23

**Authors:** Yihui Chen, Shen Zhang, Hetong Lin, Junzheng Sun, Yifen Lin, Hui Wang, Mengshi Lin, John Shi

**Affiliations:** ^1^Institute of Postharvest Technology of Agricultural Products, College of Food Science, Fujian Agriculture and Forestry University, Fuzhou, China; ^2^Food Science Program, Division of Food System & Bioengineering, University of Missouri, Columbia, MO, United States; ^3^Guelph Food Research Center, Agriculture and Agri-Food Canada, Guelph, ON, Canada

**Keywords:** longan (*Dimocarpus longan* Lour.), *Phomopsis longanae* Chi, disease development, cell wall components, cell wall-degrading enzymes, cell wall disassembly

## Abstract

The main goal of this study was to investigate the influences of *Phomopsis longanae* Chi infection on activities of cell wall-degrading enzymes (CWDEs), and contents of cell wall components in pericarp of harvested “Fuyan” longan (*Dimocarpus longan* Lour. cv. Fuyan) fruit and its relation to disease development. The results showed that, compared with the control samples, *P. longanae*-inoculated longans showed higher fruit disease index, lower content of pericarp cell wall materials (CWMs), as well as lower contents of pericarp cell wall components (chelate-soluble pectin (CSP), sodium carbonate-soluble pectin, hemicelluloses, and cellulose), but higher content of pericarp water-soluble pectin (WSP). In addition, the inoculation treatment with *P. longanae* significantly promoted the activities of CWDEs including pectinesterase, polygalacturonase, β-galactosidase, and cellulase. The results suggested that the *P. longanae* stimulated-disease development of harvested longans was due to increase in activities of pericarp CWDEs, which might accelerate the disassembly of pericarp cell wall components. In turn, resulting in the degradation of pericarp cell wall, reduction of pericarp mechanical strength, and subsequently leading to the breakdown of longan pericarp tissues. Eventually resulting in development of disease development and fruit decay in harvested longans during storage at 28°C.

## Introduction

In developed countries, fruit decay caused by pathogens affects 20–25% of the harvested fruits during post-harvest handling and storage. While in developing countries, the situation is even worse due to inadequate transportation, storage, and preservation facilities for fruits (Al-Hindi et al., 2011). Infection by pathogenic bacteria and fungi could take place in almost every step of fruit production from pre-harvest to post-harvest storage and marketing (Yao and Tian, 2005; Aghdam and Fard, 2017; Li et al., 2017).

In botanic cells, the cell wall is the first barrier against the infection by fungal pathogens (Kubicek et al., 2014). Cell wall-degrading enzymes (CWDEs) secreted by pathogens play a key role in penetrating the cell wall to utilize the nutrients (Kang and Buchenauer, 2000; Lalaoui et al., 2000; Kikot et al., 2009; Tian et al., 2009; Gharbi et al., 2015; Ramos et al., 2016). There is a diverse array of CWDEs, including polygalacturonase (PG), pectin methylgalacturonase (PMG), pectinesterase (PE), pectin lyase (PL), pectate lyase (PNL), cellulase (C_X_), β-glucosidase, and xylanases (Al-Hindi et al., 2011; Li et al., 2012; Kubicek et al., 2014). Ramos et al. (2016) found that *Macrophomina phaseolina* induced the cell wall degradation of maize and sunflower, which was initiated by the pectinase that was the first CWDE secreted by *M. phaseolina*. The activities of PG and PMG were higher than C_X_ that appeared in the later stage of the degradation process (Ramos et al., 2016). This sequence promoted the initial tissue maceration before the degradation of cell wall materials (CWMs). It was reported that *Fusarium culmorum* was able to secrete CWDEs including cellulases, xylanases, and pectinases. These CWDEs degraded the wheat spike plant tissues (cellulose, xylan, and pectin) and enabled the invasion to the tissues by *F. culmorum* (Kang and Buchenauer, 2000). Kang and Buchenauer (2000) also reported that in the cell wall, the degree of pectin degradation was higher compared with cellulose and xylan at the early stage of infection, which implied that there might be earlier secretion or higher activity of pectinases over cellulases or xylanases. Moreover, Li et al. (2012) showed that *Botryodiplodia theobromae* Pat. caused the stem-end rot of mangoes by producing PG, PMG, and C_X_ that disrupted the fruit tissues in the deterioration process. Therefore, it can be concluded that the secretion of CWDEs plays an important role in the degradation of plant cell wall during pathogenesis.

Longan is a well-known subtropical fruit with a short shelf life at room temperature due to its high susceptibility to pathogenic infections (Chen et al., 2014; Lin et al., 2017a,b,c, 2018; Zhang et al., 2017, 2018; Sun et al., 2018). Our previous studies demonstrated that *Phomopsis longanae* Chi (*P. longanae*) is one of the dominating pathogens that can cause postharvest decay of longans (Chen et al., 2011a,b, 2014; Lin et al., 2017a). To date, there has been no report on the types or activities of the CWDEs produced by *P. longanae*, and there is still a lack of research regarding the effect of these enzymes on infected longan fruits. The objective of this study was to investigate the changes of CWDEs activities during disease development in *P. longanae*-inoculated longan fruits and their effect on the degradation of cell wall. This study also aimed to elucidate the CWDEs’ function during the infection of *P. longanae* on harvested longan fruits.

## Materials and Methods

### Inoculation of Longan Fruits

*Phomopsis longanae* culturing and the preparation of spore suspension were performed as described in our previous publication (Chen et al., 2014). The concentration of spore suspension was diluted to 1 × 10^4^ spores mL^-1^ and used for inoculation.

“Fuyan” longan (*Dimocarpus longan* Lour. cv. Fuyan) fruit at commercial maturity were handpicked from a longan orchard (Quanzhou, Fujian, China). The harvested fruit were carefully packed and transported to a research laboratory in Fujian Agriculture and Forestry University (Fuzhou, Fujian, China) within 3 h and stored at 4°C. Fruit in uniform maturity and size were selected for the experiment and any rotten or damaged fruit were excluded.

The fruits were dipped in 0.5% sodium hypochlorite solution for 10 s to eliminate surface microorganisms, and then air-dried. A total of 150 fruits were employed to evaluate the properties of harvested fruits on day 0. The remaining 3000 fruits were randomly divided into two lots (1500 fruits per lot) for the control and *P. longanae*-inoculated treatment. The control group (1500 fruits) was dipped into the sterile deionized water for 5 min. The *P. longanae*-inoculated group (1500 fruits) was immersed into the *P. longanae* spore suspension (1 × 10^4^ spores mL^-1^) for 5 min. All fruits were then air dried and packed in a polyethylene bag with a thickness of 0.015 mm. Each bag contained 50 longan fruits and 30 bags were used for each treatment. The samples were then stored at 28°C with a relative humidity of 90%. For each treatment, three bags of fruit (total 150 longan fruits) were randomly selected on a daily basis during the storage period and used for the assessments of longan fruit. All the evaluations were conducted in triplicate.

### Assessment of the Index of Fruit Disease

Longan fruit disease was assessed based on our previous study (Chen et al., 2014). The lesion proportion on fruit surface of 50 individual longan fruits was measured and defined to five disease scales. The calculations of fruit disease index were performed based on the method of Chen et al. (2014).

### Preparation of CWM

Extraction of CWM was based on the modified procedures described in Duan et al. (2011) and Chen et al. (2017a,b). Ten grams of frozen longan pericarp were homogenized in 200 mL of 80 % ethanol. The mixture was boiled for 30 min with stirring. The solution was then cooled to room temperature, followed by filtration with filter papers (ϕ11 cm Medium-Speed, Whatman, Zhejiang, China). The residues were subsequently washed with 200 mL of 80% ethanol, immersed into 50 mL of 90 % dimethyl sulfoxide for 8 h to remove starches. It was subsequently washed with 200 mL of acetone, dried for 3 days at 40°C to report the final weight as CWM.

### Fractionation and Analysis of Cell Wall Components

Cell wall components fractionation and analysis were followed the procedures reported in Rugkong et al. (2010) and Chen et al. (2017a,b) with some modifications. Water-soluble pectin (WSP) was extracted via dispersing CWM (300 mg) in sodium acetate buffer (50 mmol L^-1^, pH 6.5) for 6 h with shaking (SKY-200B, SUKUN, Shanghai, China). The mixture was then centrifuged (10,000 × *g*, 4°C) for 10 min (Centrifuge 5810R, Eppendorf AG, Hamburg, Germany). The sediment was immersed in 20 mL of 50 mmol L^-1^ ethylene diamine tetraacetic acid (EDTA) with 1 mol L^-1^ NaCl (pH 6.8). After shaking and centrifugation, the supernatant containing chelate-soluble pectin (CSP) was collected. Residues were further immersed in 20 mL of 50 mmol L^-1^ Na_2_CO_3_ with 20 mmol L^-1^ NaBH_4_ for another shaking and centrifugation. The supernatant containing Na_2_CO_3_-soluble pectin (NSP) was collected. The remaining residue was further dipped in 10 mL of 4 mmol L^-1^ KOH solution with 100 mmol L^-1^ NaBH_4_ for shaking and centrifugation, and the supernatant collected was considered as hemicellulose. A volume of 1% sodium sulfite and 50 mL of 8 mol L^-1^ KOH solution with 10 mmol L^-1^ NaBH_4_ were then added to the remaining residue for shaking and centrifugation, and the supernatant containing cellulose was collected. The amounts of WSP, CSP, and NSP were determined via m-hydroxydiphenyl method (Wang et al., 2015; Chen et al., 2017a,b). The amount of hemicellulose and cellulose were determined based on the anthrone method (Wang et al., 2015; Chen et al., 2017a,b).

### Extraction and Assay of CWDEs

Enzyme extraction was based on the procedures described by Andrews and Li (1995) and Chen et al. (2017a,b). Briefly, frozen longan pericarp (1 g) was ground with 8 mL of 40 mmol L^-1^ sodium acetate buffer (containing 100 mmol L^-1^ NaCl, 2% mercaptoethanol and 5% PVP, pH 5.2). The homogenous solution was centrifuged (12,000 × *g*, 20 min) at 4°C. The collected supernatant was used to measure the activities of PE, PG, β-galactosidase and cellulase.

Pectinesterase activity was measured by combining 3 mL of crude enzyme with 10 mL of 1% pectin for the titration using 0.01 mol L^-1^ NaOH (pH 7.4 at 37°C for 30 min). The amount of enzyme that consumed 1 μmol NaOH solution per hour was used to define one unit of PE activity.

Polygalacturonase activity was determined by mixing 5 mL of 20 mmol L^-1^ sodium acetate (pH 4.0), 2 mL of 1% (*w*/*v*) polygalacturonic acid, and 1 mL of crude enzyme extract, followed by incubation at 37°C for the 30 min. A volume of 2 mL of 10 mmol L^-1^ Na_2_B_4_O_7_ was then added to the reaction mixture before terminating the reaction with 0.1 mL of 1% (*w*/*v*) 2-cyanoacetamide and boiling for 5 min. The boiled reaction mixture without adding substrate was used as the blank. The concentrations of the reducing groups were measured at 276 nm with D-galacturonic acid as the standard. The amount of enzyme producing 1 μmol galacturonic acid per hour was considered as one unit of PG activity.

To determine the β-galactosidase activity, 5 mL of 20 mmol L^-1^ sodium acetate (pH 4.7), 2 mL of 3 mmol L^-1^ *p-*nitrophenyl-β-D-galactopyranoside, and 1 mL of crude enzyme were combined and incubated at 37°C for 30 min. A volume of 2 mL of 0.2 mmol L^-1^ Na_2_CO_3_ was then added to the mixture. The concentration of the reducing product was determined at 400 nm with *p*-nitrophenol (PNP) as a standard. One unit of β-galactosidase activity referred to the amount of enzyme that produced 1 μmol PNP per hour.

Cellulase activity was assayed by mixing 5 mL of 20 mmol L^-1^ sodium acetate (pH 4.0), 1 mL of 0.25% carboxymethyl cellulose, and 1 mL of crude enzyme extract. The mixture was incubated at 37°C for 30 min followed by addition of 2 mL of 10 mmol L^-1^ Na_2_B_4_O_7_ and 0.1 mL of 1% (*w*/*v*) 2-cyanoacetamide. The reaction was stopped by heating in a boiling water bath for 5 min. A blank was prepared for each sample by boiling the reaction mixture before addition of substrate. One unit of cellulase activity referred to the amount of enzyme that produced 1 μg D-glucose per hour.

The activities of CWDEs were presented as U mg^-1^ protein. Protein content was measured following the method of Bradford (1976) using bovine serum as standard.

### Statistical Analysis

All experiments were repeated three time and data were acquired. The values in figures were expressed in the format of the mean values and standard errors. Analysis of variance (ANOVA) was used to analyze the data using the software (SPSS version 17.0). Student’s *t*-test was used to compare the mean values of the data set. A *P*-value of less than or equal to 0.05 or 0.01 was considered statistically significant.

## Results and Discussion

### Changes in Fruit Disease Index

**FIGURE 1:**
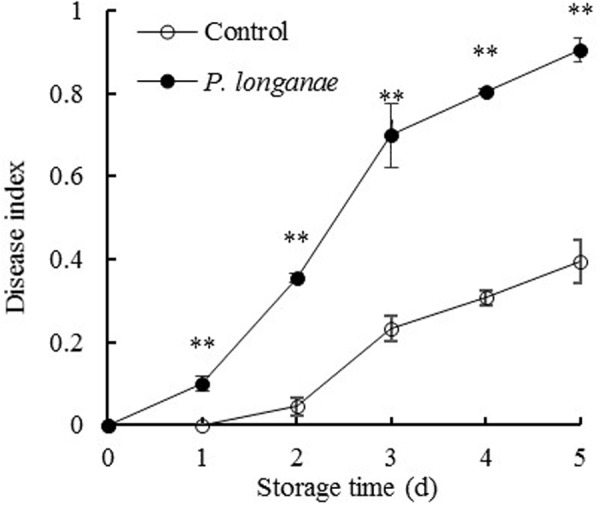
Effects of *Phomopsis longanae* infection on fruit disease index of harvested longan fruit during storage at 28°C. The asterisks indicate significant difference between control and *P. longanae*-inoculated fruit (^∗∗^*P* < 0.01). ∘, control; •, *P. longanae*-inoculation treatment.

As indicated in **Figure 1**, control fruits were intact without lesion during the first two storage days, and then the fruit disease index gradually increased with further storage. But the disease index of *P. longanae*-inoculated fruit increased rapidly throughout the storage period. Statistical analysis reveals that *P. longanae*-inoculated fruit had consistently higher fruit disease index than the control fruit at the same storage time (*P* < 0.01). After 5 days of storage, the disease index of *P. longanae*-inoculated longans was 0.9, which was almost twice as high as that of the control longans. This clearly demonstrates that inoculation treatment could significantly increase disease development of longans during storage.

### Changes in CWM

**FIGURE 2:**
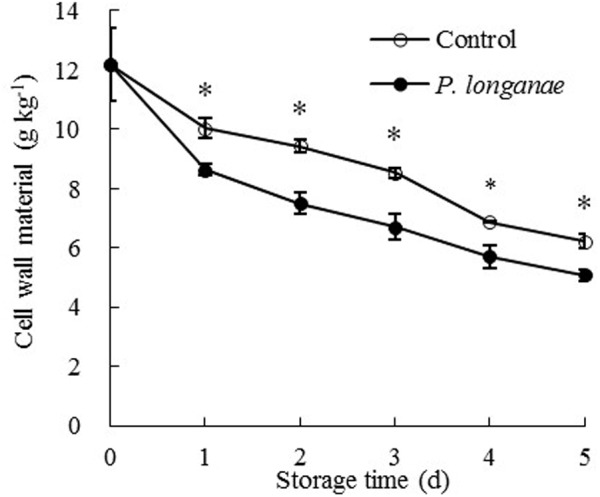
Effects of *P. longanae* infection on cell wall material (CWM) in pericarp of harvested longan fruit during storage at 28°C. The asterisks indicate significant difference between control and *P. longanae*-inoculated fruit (^∗^*P* < 0.05). ∘, control; •, *P. longanae*-inoculation treatment.

As shown in **Figure 2**, the pericarp CWM content decreased rapidly during storage and CWM readings of *P. longanae*-inoculated longans was significantly (*P* < 0.05) lower than the control longans on each storage day. After 5 days of storage, the CWM in pericarp of control longans decreased from 12.18 to 6.23 mg g^-1^, while *P. longanae*-inoculated longans has a pericarp CWM value of 5.08 mg g^-1^ on storage day 5. Correlation analysis indicates that there was a significant negative correlation between disease index (*y*) and CWM content (*x*) (*y* = 1.5173-0.1362 *x*, *r* = -0.915, *P* < 0.05) for the *P. longanae*-inoculated longans during storage. These findings indicate that the disease development or loss of disease resistance of longan fruits during storage could lead to cell wall disassembly.

### Changes in Cell Wall Components

Cell wall components including pectic substances, hemicelluloses, and cellulose constitute the material basis for the mechanical properties of cell wall, and also for maintaining the mechanical strength of the pericarp (Huang et al., 1999; Vorwerk et al., 2004). Pectic substances like WSP, CSP, and NSP are located in the primary cell wall and the middle lamella. The degradation of pectic substances led to cellulose and hemicellulose disassembly, which caused pericarp tissue loosing or fruit softening (Duan et al., 2008; Zhou et al., 2011; Chen et al., 2017a,b). In a similar study performed on litchi fruit, higher levels of structural materials like insoluble pectin, hemicellulose, and cellulose were observed in the cell walls of ‘Huaizhi’ litchi fruit pericarp compared with ‘Nuomici’ litchis, which might notably correlate to better pericarp structural strength (Huang et al., 1999).

**FIGURE 3:**
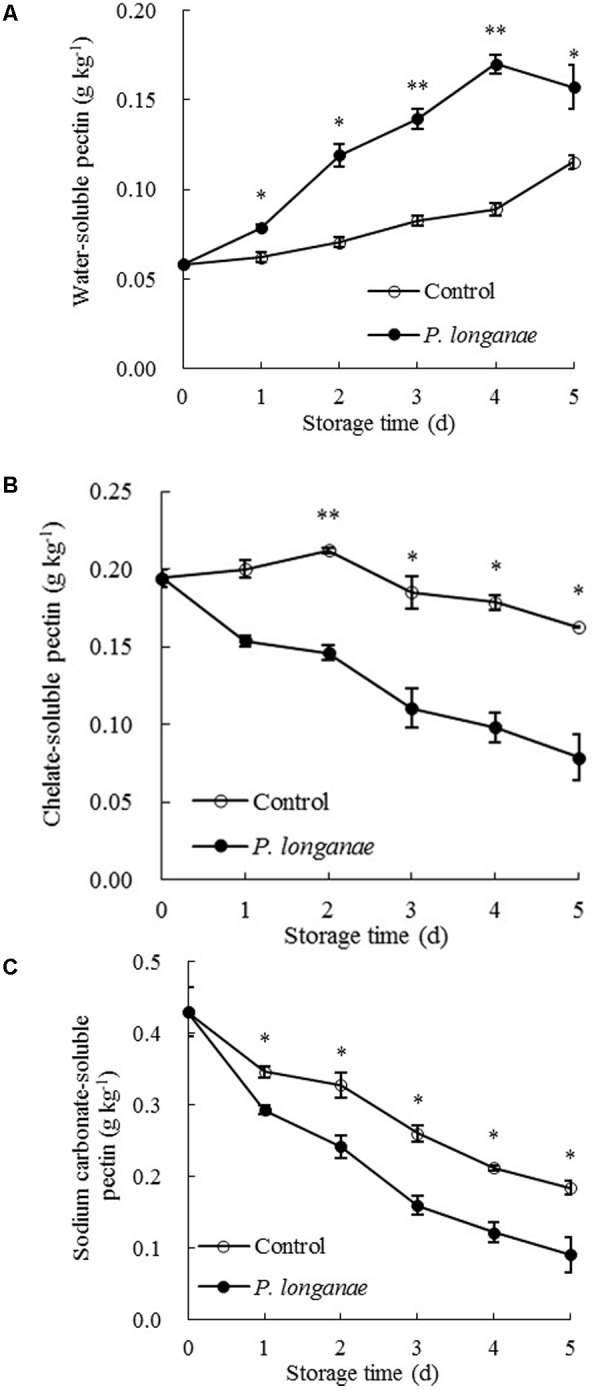
Effects of *P. longanae* infection on water-soluble pectin (WSP) **(A)**, chelate-soluble pectin (CSP) **(B)** and sodium carbonate-soluble pectin **(C)** in pericarp of harvested longan fruit during storage at 28°C. The asterisks indicate significant difference between control and *P. longanae*-inoculated fruit (^∗^*P* < 0.05, ^∗∗^*P* < 0.01). ∘, control; •, *P. longanae*-inoculation treatment.

**FIGURE 4:**
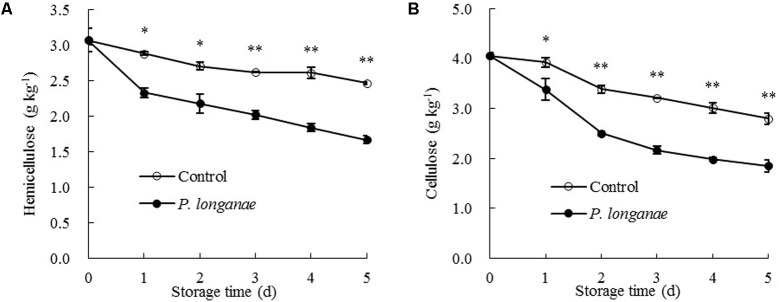
Effects of *P. longanae* infection on hemicellulose **(A)** and cellulose **(B)** in pericarp of harvested longan fruit during storage at 28°C. The asterisks indicate significant difference between control and *P. longanae*-inoculated fruit (^∗^*P* < 0.05, ^∗∗^*P* < 0.01). ∘, control; •, *P. longanae*-inoculation treatment.

The data acquired from this work indicate that *P. longanae*-inoculated longans had a faster increase in pericarp WSP than the control longans (**Figure 3A**). *P. longanae*-inoculated longans also had faster reduction in pericarp CSP contents (**Figure 3B**), NSP (**Figure 3C**), hemicellulose (**Figure 4A**), and cellulose (**Figure 4B**) than the control longans. Furthermore, fruit disease index (*y*) shows negative correlations with CSP content (*x*) (*y* = 1.6142-8.7271 *x*, *r* = -0.971, *P* < 0.01), NSP content (*x*) (*y* = 1.1252-2.9018 *x*, *r* = -0.962, *P* < 0.01), hemicellulose (*x*) (*y* = 1.0903-2.584 *x*, *r* = -0.916, *P* < 0.05) and cellulose (*x*) (*y* = 1.58-0.4145 *x*, *r* = -0.956, *P* < 0.01) in pericarp of *P. longanae*-inoculated longan fruit during storage. However, a positive correlation between fruit disease index (*y*) and WSP content (*x*) (*y* = -0.5308 + 8.3717 *x*, *r* = 0.972, *P* < 0.01) in pericarp of *P. longanae*-inoculated longan fruit was observed. In short, *P. longanae*-inoculation treatment accelerated the degradation of the cell wall components including CSP, NSP, hemicellulose, and cellulose in longans pericarp cell wall and middle lamella; however, the degraded cell wall components like WSP was elevated. Therefore, the mechanical strength of the cell wall of longan pericarp was decreased during disease development. Cell wall disassembly of longan pericarp may facilitate further pathogen invasion and dissemination.

### Changes in Cell Wall Degrading Enzymes

**FIGURE 5:**
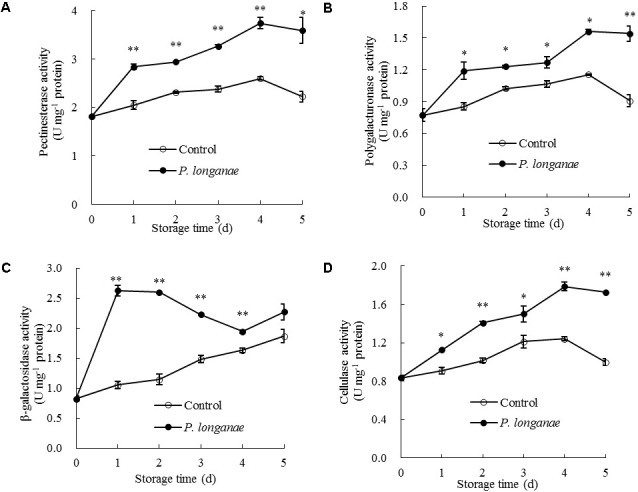
Effects of *P. longanae* infection on activities of pectinesterase **(A)**, polygalacturonase **(B)**, β-galactosidase **(C)**, and cellulase **(D)** in pericarp of harvested longan fruit during storage at 28°C. The asterisks indicate significant difference between control and *P. longanae*-inoculated fruit (^∗^*P* < 0.05, ^∗∗^*P* < 0.01). ∘, control; •, *P. longanae*-inoculation treatment.

To further explain the change of cell wall components during disease development, changes in CWDEs were measured. PE and PG activities in pericarp of control fruit rose gradually toward the maximum on day 4 and decreased afterwards (**Figures 5A,B**). The PE and PG activities in pericarp of *P. longanae*-inoculated longans followed a similar trend as the control longans but had significantly (*P* < 0.05) higher activities (**Figures 5A,B**). It has been reported that the softening of pericarp tissues were associated with the changes in pectic substances, which could be attributed to the action of PE and PG (Liu et al., 2006). Specifically, PE can remove the methoxyl groups and catalyze the decomposition of galacturonic acid polymer to polygalacturonic acid, which enables PG to hydrolyze 1, 4-α-D-galacturonic bond of polygalacturonic acid to generate galacturonic acid (Liu et al., 2006; Lin et al., 2007; Rugkong et al., 2010; Wei et al., 2010). The degradation of pectic substances by the joint action of PE and PG can destroy the structure of middle lamella and decrease the mechanical strength of cell wall (Deng et al., 2005; Liu et al., 2006). In the present work, PE and PG activities increased significantly after inoculation treatment, which promoted the depolymerization and dissolution of pectin.

As indicated in **Figure 5C**, β-galactosidase activity in pericarp of *P. longanae*-inoculated longans exhibited a sharp increase on the first day of storage, then changed mildly on the second storage day, followed by a quick decline from days 2 to 4, and a rapid increase on day 5. However, the β-galactosidase activity in pericarp of the control longans increased steadily with progressing storage time, with significantly (*P* < 0.01) lower level in contrast to that of *P. longanae*-inoculated fruit from day 1 to day 4 (**Figure 5C**). β-galactosidase also plays a key role in the depolymerization and dissolution of pectic substances in fruits. It can hydrolyze β-1, 4-galactan bonds and separate galactosyl residues from pectin side chains, which may trigger some adverse reactions such as the production of ethylene and stress reaction, and thus further accelerate the disruption of cell wall structure (Liu et al., 2006; Lin et al., 2007; Wei et al., 2010; Chen et al., 2017a,b). The results of this study suggest that higher levels of β-galactosidase activity in inoculated fruits during storage contributed to the degradation of pectin polysaccharides.

Cellulose activity increased gradually in both control and *P. longanae*-inoculated longans during storage days 1–4 and then decreased (**Figure 5D**). After 5 days of storage, *P. longanae*-inoculated longans showed 72.5% higher value of pericarp cellulose activity as compared with the control longans. Cellulase is a multi-enzyme system including endo-1, 4-β-D-glucanase, exo-1, 4-β-D-glucanase, and β-1, 4-glucosidase (Lin et al., 2007). Cellulase could cause the degradation of cellulose and xyloglucan in cell wall structure, which resulted in pericarp tissue loosing and fruit softening (Deng et al., 2005; Liu et al., 2006; Zhou et al., 2011; Bu et al., 2013; Chen et al., 2017a,b). In this study, the enhanced activity of cellulase due to the *P. longanae*-inoculation treatment correlated well with cellulose degradation (**Figure 4B**) in pericarp of longan fruit during storage.

## Conclusion

In summary, as compared with the control, fruit inoculated with *P. longanae* could lead to significantly higher fruit disease index, increased activity of CWDEs (e.g., PE, PG, β-galactosidase and cellulose), and lower levels of CWM and cell wall components (such as CSP, NSP, hemicelluloses, and cellulose) in pericarp of harvested longan fruit. These results indicate that *P. longanae* infection can accelerate the cell wall degradation of longan pericarp during disease development by promoting CWDEs activities, which decreased the mechanical strength of the cell walls, resulting in longan pericarp tissue softening, and eventually leading to fruit decay.

## Author Contributions

YC and HL designed the research. SZ, JZS, YL, and HW conducted the experiments and analyzed the data. YC and SZ wrote the manuscript. HL revised the manuscript. ML and JS edited English language of the manuscript. All authors approved the submission and publication of the manuscript.

## Conflict of Interest Statement

The authors declare that the research was conducted in the absence of any commercial or financial relationships that could be construed as a potential conflict of interest.

## References

[B1] Aghdam M. S. Fard J. R. (2017). Melatonin treatment attenuates postharvest decay and maintains nutritional quality of strawberry fruits (*Fragaria* × *anannasa* cv. Selva) by enhancing GABA shunt activity. 221 1650–1657. 10.1016/j.foodchem.2016.10.123 27979142

[B2] Al-Hindi R. R. Al-Najada A. R. Mohamed S. A. (2011). Isolation and identification of some fruit spoilage fungi: screening of plant cell wall degrading enzymes. 5 443–448. 10.5897/AJMR10.896

[B3] Andrews P. K. Li S. L. (1995). Cell wall hydrolytic enzyme activity development of nonclimacteric sweet cherry (*Prunus avium* L.) fruit. 70 561–567. 10.1080/14620316.1995.11515327

[B4] Bradford M. M. (1976). A rapid and sensitive method for the quantitation of microgram quantities of protein utilizing the principle of protein-dye binding. 72 248–254. 10.1016/0003-2697(76)90527-3 942051

[B5] Bu J. W. Yu Y. C. Aisikaer G. Ying T. J. (2013). Postharvest UV-C irradiation inhibits the production of ethylene and the activity of cell wall-degrading enzymes during softening of tomato (*Lycopersicon esculentum* L.) fruit. 86 337–345. 10.1016/j.postharvbio.2013.07.026

[B6] Chen Y. H. Hung Y. C. Chen M. Y. Lin H. T. (2017a). Effects of acidic electrolyzed oxidizing water on retarding cell wall degradation and delaying softening of blueberries during postharvest storage. 84 650–657. 10.1016/j.lwt.2017.06.011

[B7] Chen Y. H. Sun J. Z. Lin H. T. Hung Y. C. Zhang S. Lin Y. F. (2017b). Paper-based 1-MCP treatment suppresses cell wall metabolism and delays softening of Huanghua pears during storage. 97 2547–2552. 10.1002/jsfa.8072 27706823

[B8] Chen Y. H. Lin H. T. Jiang Y. M. Zhang S. Lin Y. F. Wang Z. H. (2014). *Phomopsis longanae* Chi-induced pericarp browning and disease development of harvested longan fruit in association with energy status. 93 24–28. 10.1016/j.postharvbio.2014.02.003

[B9] Chen Y. H. Lin H. T. Lin Y. F. Zhang J. N. Zhao Y. F. (2011a). Effects of *Phomopsis longanae* Chi infection on browning and active oxygen metabolism in pericarp of harvested longan fruits. 44 4858–4866. 10.3864/j.issn.0578-1752.2011.23.012

[B10] Chen Y. H. Lin H. T. Lin Y. F. Zhao Y. F. Zhang J. N. (2011b). Effects of *Phomopsis longanae* Chi infection on lipoxygenase activity and fatty acid constituents of membrane lipids in pericarp of harvested longan fruits. 19 260–266. 10.3969/j.issn.1005-3395.2011.03.011

[B11] Deng Y. Wu Y. Li Y. F. (2005). Changes in firmness, cell wall composition and cell wall hydrolases of grapes stored in high oxygen atmospheres. 38 769–776. 10.1016/j.foodres.2005.03.003

[B12] Duan X. W. Cheng G. P. Yang E. Yi C. Ruenroengklin N. Lu W. J. (2008). Modification of pectin polysaccharides during ripening of postharvest banana fruit. 111 144–149. 10.1016/j.foodchem.2008.03.049

[B13] Duan X. W. Zhang H. Y. Zhang D. D. Sheng J. F. Lin H. T. Jiang Y. M. (2011). Role of hydroxyl radical in modification of cell wall polysaccharides and aril breakdown during senescence of harvested longan fruit. 128 203–207. 10.1016/j.foodchem.2011.03.031 25214349

[B14] Gharbi Y. Alkher H. Triki M. A. Barkallah M. Emna B. Trabelsi R. (2015). Comparative expression of genes controlling cell wall-degrading enzymes in *Verticillium dahliae* isolates from olive, potato and sunflower. 91 56–65. 10.1016/j.pmpp.2015.05.006

[B15] Huang X. M. Wang H. C. Gao F. F. Huang H. B. (1999). A comparative study of the pericarp of litchi cultivars susceptible and resistant to fruit cracking. 74 351–354. 10.1080/14620316.1999.11511120

[B16] Kang Z. Buchenauer H. (2000). Ultrastructural and cytochemical studies on cellulose, xylan and pectin degradation in wheat spikes infected by *Fusarium culmorum*. 148 263–275. 10.1046/j.1439-0434.2000.00489.x

[B17] Kikot G. E. Hours R. A. Alconada T. M. (2009). Contribution of cell wall degrading enzymes to pathogenesis of *Fusarium graminearum*: a review. 49 231–241. 10.1002/jobm.200800231 19025875

[B18] Kubicek C. P. Starr T. L. Glass N. L. (2014). Plant cell wall-degrading enzymes and their secretion in plant-pathogenic fungi. 52 427–451. 10.1146/annurev-phyto-102313-045831 25001456

[B19] Lalaoui F. Halama P. Dumortier V. Paul B. (2000). Cell wall-degrading enzymes produced in vitro by isolates of *Phaeosphaeria nodorum* differing in aggressiveness. 49 727–733. 10.1046/j.1365-3059.2000.00491.x

[B20] Li J. K. Lei H. H. Song H. M. Lai T. F. Xu X. B. Shi X. Q. (2017). 1-methylcyclopropene (1-MCP) suppressed postharvest blue mold of apple fruit by inhibiting the growth of *Penicillium expansum*. 125 59–64. 10.1016/j.postharvbio.2016.11.005

[B21] Li M. Gao Z. Y. Hu M. J. Zhou S. Yang D. P. Yang B. (2012). Pathogenicity of cell wall degrading enzymes produced by *Botryodiplodia theobromae* Pat. against mangoes. 1 18–21.

[B22] Lin H. T. Zhao Y. F. Xi Y. F. (2007). Changes in cell wall components and cell wall-degrading enzyme activities of postharvest longan fruit during aril breakdown. 33 137–145. 10.3321/j.issn:1671-3877.2007.02.007 17452799

[B23] Lin Y. F. Chen M. Y. Lin H. T. Hung Y. C. Lin Y. X. Chen Y. H. (2017a). DNP and ATP induced alteration in disease development of *Phomopsis longanae* Chi-inoculated longan fruit by acting on energy status and reactive oxygen species production-scavenging system. 228 497–505. 10.1016/j.foodchem.2017.02.045 28317755

[B24] Lin Y. F. Lin Y. X. Lin H. T. Chen Y. H. Wang H. Shi J. (2018). Application of propyl gallate alleviates pericarp browning in harvested longan fruit by modulating metabolisms of respiration and energy. 240 863–869. 10.1016/j.foodchem.2017.07.118 28946353

[B25] Lin Y. F. Lin Y. X. Lin H. T. Ritenour M. A. Shi J. Zhang S. (2017b). Hydrogen peroxide-induced pericarp browning of harvested longan fruit in association with energy metabolism. 225 31–36. 10.1016/j.foodchem.2016.12.088 28193430

[B26] Lin Y. F. Lin Y. X. Lin H. T. Shi J. Chen Y. H. Wang H. (2017c). Inhibitory effects of propyl gallate on membrane lipids metabolism and its relation to increasing storability of harvested longan fruit. 217 133–138. 10.1016/j.foodchem.2016.08.065 27664618

[B27] Liu X. D. Wu Z. X. Han D. M. Chen W. X. Su M. X. (2006). Changes of the cell-wall metabolism enzymes of pericarp of longan under Storage. 2 24–28. 10.3969/j.issn.1000-2561.2006.02.005

[B28] Ramos A. M. Gally M. Szapiro G. Itzcovich T. Carabajal M. Levin L. (2016). *In vitro* growth and cell wall degrading enzyme production by Argentinean isolates of *Macrophomina phaseolina*, the causative agent of charcoal rot in corn. 48 267–273. 10.1016/j.ram.2016.06.002 27825736

[B29] Rugkong A. Rose J. K. C. Lee S. J. Giovannoni J. J. O’Neill M. A. Watkins C. B. (2010). Cell wall metabolism in cold-stored tomato fruit. 57 106–113. 10.1016/j.postharvbio.2010.03.004

[B30] Sun J. Z. Lin H. T. Zhang S. Lin Y. F. Wang H. Lin M. S. (2018). The roles of ROS production-scavenging system in *Lasiodiplodia theobromae* (Pat.) Griff. & Maubl.-induced pericarp browning and disease development of harvested longan fruit. 247 16–22. 10.1016/j.foodchem.2017.12.017 29277223

[B31] Tian C. M. Zhao P. Cao Z. M. (2009). Role of cell wall degrading enzymes in the interaction of poplar and *Melampsora larici-populina* Kleb. 4:111 10.1007/s11461-009-0006-6

[B32] Vorwerk S. Somerville S. Somerville C. (2004). The role of plant cell wall polysaccharide composition in disease resistance. 4 203–209. 10.1016/j.tplants.2004.02.005 15063871

[B33] Wang L. Jin P. Wang J. Jiang L. L. Shan T. M. Zheng Y. H. (2015). Effect of β-aminobutyric acid on cell wall modification and senescence in sweet cherry during storage at 20°C. 175 471–477. 10.1016/j.foodchem.2014.12.011 25577108

[B34] Wei J. M. Ma F. W. Shi S. G. Qi X. D. Zhu X. Q. Yuan J. W. (2010). Changes and postharvest regulation of activity and gene expression of enzymes related to cell wall degradation in ripening apple fruit. 56 147–154. 10.1016/j.postharvbio.2009.12.003

[B35] Yao H. J. Tian S. P. (2005). Effects of pre- and post-harvest application of salicylic acid or methyl jasmonate on inducing disease resistance of sweet cherry fruit in storage. 35 253–262. 10.1016/j.postharvbio.2004.09.001

[B36] Zhang S. Lin H. T. Lin Y. F. Lin Y. X. Hung Y. C. Chen Y. H. (2017). Energy status regulates disease development and respiratory metabolism of *Lasiodiplodia theobromae* (Pat.) Griff. & Maubl.-infected longan fruit. 231 238–246. 10.1016/j.foodchem.2017.03.132 28450002

[B37] Zhang S. Lin Y. Z. Lin H. T. Lin Y. X. Chen Y. H. Wang H. (2018). *Lasiodiplodia theobromae* (Pat.) Griff. & Maubl.-induced disease development and pericarp browning of harvested longan fruit in association with membrane lipids metabolism. 244 93–101. 10.1016/j.foodchem.2017.10.020 29120810

[B38] Zhou R. Li Y. F. Yan L. P. Xie J. (2011). Effect of edible coatings on enzymes, cell-membrane integrity, and cell-wall constituents in relation to brittleness and firmness of Huanghua pears (*Pyrus pyrifolia* Nakai, cv. Huanghua) during storage. 124 569–575. 10.1016/j.foodchem.2010.06.075

